# Supplementary material for: HIVprotI: an integrated web based platform for prediction and design of HIV proteins inhibitors
Source: J Cheminform. 2018 Mar 9;10:12. doi: 10.1186/s13321-018-0266-y (PMC5845081; doi:10.1186/s13321-018-0266-y)
Supplement: Supplementary file 2 — Additional file 2. Source code of HIVProtI web server. [file 13321_2018_266_MOESM2_ESM.zip › HIVprotI_Source-code/hivprotI/weka-3-6-6/doc/weka/experiment/CostSensitiveClassifierSplitEvaluator.html]

CostSensitiveClassifierSplitEvaluator


---


|  |  |  |
| --- | --- | --- |
| **Overview** \| **Package** \| **Class** \| **Tree** \| **Deprecated** \| **Index** \| **Help** \| **Weka's home** | |  |
| **PREV CLASS**   **NEXT CLASS** | **FRAMES**    **NO FRAMES**     **All Classes** |  |
| SUMMARY: NESTED \| FIELD \| CONSTR \| METHOD | DETAIL: FIELD \| CONSTR \| METHOD |  |


---


## weka.experiment Class CostSensitiveClassifierSplitEvaluator

```
java.lang.Object
  weka.experiment.ClassifierSplitEvaluator
      weka.experiment.CostSensitiveClassifierSplitEvaluator
```

**All Implemented Interfaces:**: java.io.Serializable, AdditionalMeasureProducer, OptionHandler, RevisionHandler, SplitEvaluator

---

``` public class CostSensitiveClassifierSplitEvaluator extends ClassifierSplitEvaluator ```

SplitEvaluator that produces results for a classification scheme on a nominal class attribute, including weighted misclassification costs.


Valid options are:

```
 -W <class name>
  The full class name of the classifier.
  eg: weka.classifiers.bayes.NaiveBayes
```

```
 -C <index>
  The index of the class for which IR statistics
  are to be output. (default 1)
```

```
 -I <index>
  The index of an attribute to output in the
  results. This attribute should identify an
  instance in order to know which instances are
  in the test set of a cross validation. if 0
  no output (default 0).
```

```
 -P
  Add target and prediction columns to the result
  for each fold.
```

```
 Options specific to classifier weka.classifiers.rules.ZeroR:
```

```
 -D
  If set, classifier is run in debug mode and
  may output additional info to the console
```

```
 -D <directory>
  Name of a directory to search for cost files when loading
  costs on demand (default current directory).
```

All options after -- will be passed to the classifier.

**Version:**
:   $Revision: 7516 $

**Author:**
:   Len Trigg (len@reeltwo.com)

**See Also:**: Serialized Form

---

**Constructor Summary**

`CostSensitiveClassifierSplitEvaluator()`


| **Method Summary** | |
| --- | --- |
| `java.io.File` | `getOnDemandDirectory()`             Returns the directory that will be searched for cost files when loading on demand. |
| `java.lang.String[]` | `getOptions()`             Gets the current settings of the Classifier. |
| `java.lang.Object[]` | `getResult(Instances train, Instances test)`             Gets the results for the supplied train and test datasets. |
| `java.lang.String[]` | `getResultNames()`             Gets the names of each of the result columns produced for a single run. |
| `java.lang.Object[]` | `getResultTypes()`             Gets the data types of each of the result columns produced for a single run. |
| `java.lang.String` | `getRevision()`             Returns the revision string. |
| `java.lang.String` | `globalInfo()`             Returns a string describing this split evaluator |
| `java.util.Enumeration` | `listOptions()`             Returns an enumeration describing the available options.. |
| `java.lang.String` | `onDemandDirectoryTipText()`             Returns the tip text for this property |
| `void` | `setOnDemandDirectory(java.io.File newDir)`             Sets the directory that will be searched for cost files when loading on demand. |
| `void` | `setOptions(java.lang.String[] options)`             Parses a given list of options. |
| `java.lang.String` | `toString()`             Returns a text description of the split evaluator. |

**Methods inherited from class weka.experiment.ClassifierSplitEvaluator**

`classifierTipText, enumerateMeasures, getAttributeID, getClassForIRStatistics, getClassifier, getKey, getKeyNames, getKeyTypes, getMeasure, getPredTargetColumn, getRawResultOutput, setAdditionalMeasures, setAttributeID, setClassForIRStatistics, setClassifier, setClassifierName, setPredTargetColumn`

**Methods inherited from class java.lang.Object**

`equals, getClass, hashCode, notify, notifyAll, wait, wait, wait`

**Constructor Detail**

### CostSensitiveClassifierSplitEvaluator

```
public CostSensitiveClassifierSplitEvaluator()
```


**Method Detail**

### globalInfo

```
public java.lang.String globalInfo()
```

:   Returns a string describing this split evaluator

    :   **Overrides:**: `globalInfo` in class `ClassifierSplitEvaluator`
    :   **Returns:**: a description of the split evaluator suitable for displaying in the explorer/experimenter gui

---


### listOptions

```
public java.util.Enumeration listOptions()
```

:   Returns an enumeration describing the available options..

    :   **Specified by:**: `listOptions` in interface `OptionHandler` **Overrides:**: `listOptions` in class `ClassifierSplitEvaluator`
    :   **Returns:**: an enumeration of all the available options.

---


### setOptions

```
public void setOptions(java.lang.String[] options)
                throws java.lang.Exception
```

:   Parses a given list of options.


    Valid options are:

    ```
     -W <class name>
      The full class name of the classifier.
      eg: weka.classifiers.bayes.NaiveBayes
    ```

    ```
     -C <index>
      The index of the class for which IR statistics
      are to be output. (default 1)
    ```

    ```
     -I <index>
      The index of an attribute to output in the
      results. This attribute should identify an
      instance in order to know which instances are
      in the test set of a cross validation. if 0
      no output (default 0).
    ```

    ```
     -P
      Add target and prediction columns to the result
      for each fold.
    ```

    ```
     Options specific to classifier weka.classifiers.rules.ZeroR:
    ```

    ```
     -D
      If set, classifier is run in debug mode and
      may output additional info to the console
    ```

    ```
     -D <directory>
      Name of a directory to search for cost files when loading
      costs on demand (default current directory).
    ```

    All options after -- will be passed to the classifier.

    :   **Specified by:**: `setOptions` in interface `OptionHandler` **Overrides:**: `setOptions` in class `ClassifierSplitEvaluator`
    :   **Parameters:**: `options` - the list of options as an array of strings **Throws:**: `java.lang.Exception` - if an option is not supported

---


### getOptions

```
public java.lang.String[] getOptions()
```

:   Gets the current settings of the Classifier.

    :   **Specified by:**: `getOptions` in interface `OptionHandler` **Overrides:**: `getOptions` in class `ClassifierSplitEvaluator`
    :   **Returns:**: an array of strings suitable for passing to setOptions

---


### onDemandDirectoryTipText

```
public java.lang.String onDemandDirectoryTipText()
```

:   Returns the tip text for this property

    :   **Returns:**: tip text for this property suitable for displaying in the explorer/experimenter gui

---


### getOnDemandDirectory

```
public java.io.File getOnDemandDirectory()
```

:   Returns the directory that will be searched for cost files when
    loading on demand.

    :   **Returns:**: The cost file search directory.

---


### setOnDemandDirectory

```
public void setOnDemandDirectory(java.io.File newDir)
```

:   Sets the directory that will be searched for cost files when
    loading on demand.

    :   **Parameters:**: `newDir` - The cost file search directory.

---


### getResultTypes

```
public java.lang.Object[] getResultTypes()
```

:   Gets the data types of each of the result columns produced for a
    single run. The number of result fields must be constant
    for a given SplitEvaluator.

    :   **Specified by:**: `getResultTypes` in interface `SplitEvaluator` **Overrides:**: `getResultTypes` in class `ClassifierSplitEvaluator`
    :   **Returns:**: an array containing objects of the type of each result column. The objects should be Strings, or Doubles.

---


### getResultNames

```
public java.lang.String[] getResultNames()
```

:   Gets the names of each of the result columns produced for a single run.
    The number of result fields must be constant
    for a given SplitEvaluator.

    :   **Specified by:**: `getResultNames` in interface `SplitEvaluator` **Overrides:**: `getResultNames` in class `ClassifierSplitEvaluator`
    :   **Returns:**: an array containing the name of each result column

---


### getResult

```
public java.lang.Object[] getResult(Instances train,
                                    Instances test)
                             throws java.lang.Exception
```

:   Gets the results for the supplied train and test datasets. Now performs
    a deep copy of the classifier before it is built and evaluated (just in case
    the classifier is not initialized properly in buildClassifier()).

    :   **Specified by:**: `getResult` in interface `SplitEvaluator` **Overrides:**: `getResult` in class `ClassifierSplitEvaluator`
    :   **Parameters:**: `train` - the training Instances.: `test` - the testing Instances. **Returns:**: the results stored in an array. The objects stored in the array may be Strings, Doubles, or null (for the missing value). **Throws:**: `java.lang.Exception` - if a problem occurs while getting the results

---


### toString

```
public java.lang.String toString()
```

:   Returns a text description of the split evaluator.

    :   **Overrides:**: `toString` in class `ClassifierSplitEvaluator`
    :   **Returns:**: a text description of the split evaluator.

---


### getRevision

```
public java.lang.String getRevision()
```

:   Returns the revision string.

    :   **Specified by:**: `getRevision` in interface `RevisionHandler` **Overrides:**: `getRevision` in class `ClassifierSplitEvaluator`
    :   **Returns:**: the revision


---


|  |  |  |
| --- | --- | --- |
| **Overview** \| **Package** \| **Class** \| **Tree** \| **Deprecated** \| **Index** \| **Help** \| **Weka's home** | |  |
| **PREV CLASS**   **NEXT CLASS** | **FRAMES**    **NO FRAMES**     **All Classes** |  |
| SUMMARY: NESTED \| FIELD \| CONSTR \| METHOD | DETAIL: FIELD \| CONSTR \| METHOD |  |


---
